# The conspiratorial style in lay economic thinking

**DOI:** 10.1371/journal.pone.0171238

**Published:** 2017-03-03

**Authors:** David Leiser, Nofar Duani, Pascal Wagner-Egger

**Affiliations:** 1 Department of Psychology, Ben Gurion University, Beer Sheva, Israel; 2 Department of Economics, Ben Gurion University, Beer Sheva, Israel; 3 Department of Psychology, University of Fribourg, Fribourg, Switzerland; Universitat Wien, AUSTRIA

## Abstract

This study investigates patterns of lay perception of economics, and in particular the place of conspiratorial thinking regarding the economic domain. We devised four types of accounts in the economic domain, over a range of questions regarding different aspects of the economy: the classical neo-liberal economic view (which we labeled *Econ101*), and the *Conspiracy* view (the destructive outcomes of economy are due to small and powerful groups who are manipulating the markets), to which we added the *Government malfunction* view (failures in the economy are due to the authorities), and the *Bad Invisible Hand* view (the invisible hand may go wrong, and the equilibrium reached by its doings may be undesirable). The last two views are the ones most strongly endorsed by our respondents, in the US, Israel and Switzerland. The pattern of inter-correlations between the four accounts, and that between each and the psycho-social variables we examined, exhibits two clusters, *Econ101* vs. the other three views of economy. This corresponds to a general opposition between people who trust the neoliberal economic system, and those opposed to it. What sets economic conspiratorial thinking apart are its links with other conspirational beliefs and with paranormal beliefs.

## Introduction

The study of lay understanding of economics is of major practical significance. Economic beliefs affect economic behavior [1, 2] and constitute an important component of economic modelling [3]. Moreover, democratic functioning assumes that citizens understand the issues [4, 5], enabling them to affect public policy through the political process [6].

People have little understanding of such matters as the reasons of tax increases, the argument for privatization of public resources, the causes of unemployment and inflation, or the role of commercial banks (see e.g.[6–9]). Yet in this domain, as in many others, laypeople are unaware of their ignorance. "We have very little idea of how little we know. We're not designed to know how little we know" (p. 24) observes Kahneman [10]. Laypeople will pronounce themselves confidently about economic issues they poorly understand [7]. But, if they don't understand the issues, what brings them to endorse and express a particular opinion? Part of the answer is political. Political affiliation involves a set of beliefs about economics. Other explanations, discussed in [9], involve the use of metaphors and heuristics. Finally, there is the possibility that economic beliefs are influenced by socio-psychological traits or other kinds of beliefs (e.g., paranormal, conspiracy theories beliefs). This is the approach we adopt here.

A study of how lay people account for the financial crisis [11] brought to light two patterns of answers, the Liberal and the Individual. Those fitting the Liberal profile endorse the neo-liberal analysis of economics. Respondents matching the Individual profile were more critical and accusatory, blaming a range of actors for their alleged moral, cognitive, or character failings (see also [12]). The authors found that people living in the Western world, who were unaffected by the crisis, and who had benefited from economic training tend to exhibit the Liberal profile.

In this study, we aim to refine the analysis in terms of the Liberal versus the Individual views, as there is more than one way to reject the Liberal view. To delve more deeply into the alternatives, we asked our respondents to rate their agreement with four accounts of how the economy plays out: the liberal economic view, and three ways in which that model may fail to describe the functioning of the economic world. Specifically, one account presented the possibility that the invisible hand may go wrong and that the equilibrium reached by its doings is undesirable. According to this view, the liberal economic view is unfounded and unrealistically optimistic. Another relates to the possibility that the government malfunctions. The last attributes destructive outcomes to hidden sinister forces who manipulate the system for their purposes. The latter view is far from unusual. Oliver and Wood [13] showed that, in the US, half the public endorses at least one conspiratorial narrative. For the sake of generality, we ran our experiments in three Western countries, Switzerland, Israel and the US, since some authors hold that Americans have a special relationship to conspiracy theories [14, 15].

### Conspiracy theories (CTs) in psychology

CTs have been defined as unverified claims of conspiracy (which in turn may be viewed as a secret agreement between powerful individuals or organizations to hide a dishonest, immoral, or illegal act or situation) with sensationalistic subject matter or implications [16, 17]. CTs may be preferred to the official version of the political or social event or situation at stake, even though at the epistemic level they often present low standards of evidence. CTs typically involve interpreting *errant data* [18], namely unaccounted-for and contradictory elements of the official version, as evidence of a conspiracy rather than as inevitable anomalies of wide-ranging events and investigations.

Research on the social and psychological mechanisms of conspiracist ideation has been on the rise in the past years. Numerous personality factors, emotional states and social political attitudes have been found to correlate with support for CTs, including *anomia* (broadly: distrust towards authorities, feelings of powerlessness, and feelings of dissatisfaction about one’s life; [19–28], personal and social (i.e., feeling of insecurity) *anxiety* [29–31], *negative self-esteem* [19, 22, 26, 32], *paranoia* and *schizotypy* [16, 21, 26, 28, 33–37], *Right-Wing Authoritarianism* [19, 28, 32, 38]; but only marginally in [39], and *irrationality* or *paranormal beliefs* [16, 21, 24, 26, 28, 30, 35, 40–44]. Motivational processes have also been underlined. A motivation to restore a sense of certainty and a sense of control [45–47] would favor conspiracist ideation, as well as a stronger need for uniqueness [48]. Collectively, these findings suggest that the conspiracist mindset has some functional value, at least for some individuals. CTs may serve important social and cultural functions such as making sense of ambiguous, threatening events [49], as do social representations in general [50]. Conspiracist features also serve to enhance the narrative construction of the description of important events: when asked to produce a coherent story of the 9/11 terrorist attacks on Manhattan, the participants mixed the official version and CT elements in idiosyncratic and original ways [51]. Moreover, beliefs in CTs can be viewed as a political attitude *per se*, distinct from classical ones (conservatism, social dominance, etc.) [39].

## Method

### Participants

Participants from three different countries replied to an online survey (N = 289, 65% female, USA- 101; Israel- 128; Switzerland- 60). Due to the length of the questionnaire, we split some parts to different sub-groups. The detail of how many participants from each country answered each question is to be found in the Supplementary Materials file S4 Table. The study was approved by the ethics committee of the psychology department at Ben Gurion University of the Negev. Respondents were recruited through social media, non-specific online forums and Mechanical Turk (MTurk). Respondents recruited through MTurk received payment for their participation (0.75$), the remaining participants volunteered. The Swiss sample was composed of first-year psychology students (age: *M* = 22.01, *SD* = 3.26; gender: 82% female), who were given course credits for their participation. The demographic characteristics for the Israel and USA samples are summarized in Table 1 (the Swiss sample was homogenous, with high educational level, SES, and no formal economic training).

**Table 1:** Socio-demographic characteristics of the Israel and USA samples.

Item		Total • (*n* = 229)	USA • (*n* = 101)	Israel • (*n* = 128)
Age	Mean	35.24	37.59	33.39
	SD	12.36	11.35	12.84
Educational level	Less than high-school	3.93%	0.99%	6.25%
	High-school or equivalent	20.09%	34.65%	8.59%
	Associate degree	10.48%	13.86%	7.81%
	Bachelor degree	49.34%	38.61%	57.81%
	Graduate degree	16.16%	11.88%	19.53%
Religion	Religious	16.16%	25.74%	8.59%
	Traditional	18.34%	18.81%	17.97%
	Non-Religious/ Atheist	65.50%	55.45%	73.44%
SES	Low	9.61%	11.88%	7.81%
	mid-low	31.00%	42.57%	21.88%
	Mid	43.67%	41.58%	45.31%
	mid-high	12.66%	3.96%	19.53%
	High	3.06%	0.00%	5.47%
Economic training	No formal economic training	53.28%	47.52%	57.81%
	High-school economics	12.66%	20.79%	6.25%
	After H.S economics course	20.96%	27.72%	15.63%
	Full or partial degree	13.10%	3.96%	20.31%

### Materials and procedure

The online survey opened with a standard Informed Consent form, that included a line to the effect that there are no correct or wrong answers. The survey itself was comprised of two sections. In the first, respondents were asked to reply to several questions measuring personality traits and attitudes, such as Openness, Agreeableness, Irrationality, Anomie, Right-Wing Authoritarianism, Belief in a Dangerous World, and Internal Locus of Control. Participants were also asked about their beliefs regarding three well known, non-economic conspiracies (about the deaths of John F. Kennedy and Lady Diana, and the first Apollo mission on the moon), to report the extent to which they were personally affected by the 2008 financial recession, and about their views on what happened to the money that "disappeared" in the course of the financial crisis. The second part of the survey measured participants' lay perception of economics.

#### Psycho-social scales

All the questions were based on existing scales, and translated and adapted to the appropriate languages (the wording of the items in English may be found in S1 Table).

*Abbreviated 10-item Big Five questionnaire*. We used an abbreviated scale [52] that was shown to provide a short yet reliable assessment of the Big Five personality factors. As previous literature found correlations with only two of the five personality traits, agreeableness and openness, we chose to focus solely on these two dimensions (each measured by two items). Ratings were made on a 6-point scale (1 = strongly disagree, 6 = strongly agree), reversed items were adjusted and personality factors were computed by averaging related items. The standardized Cronbach *α* for openness was 0.64 (*N* = 238). The standardized Cronbach *α* for agreeableness was only 0.36 (*N* = 238), and we therefore did not include this variable in the statistical analyses.

*Right-Wing Authoritarianism*. RWA consists of three attitudinal clusters: authoritarian submission, authoritarian aggression, and conventionalism. For this measure we used the English translation of the 12 German RWA^3^D-Items from [53], and translated it to Hebrew and French (for the Israeli and Swiss samples). Native language speakers checked the equivalence between the translations and the original formulation. Ratings were made on a 6-point scale (1 = strongly disagree, 6 = strongly agree) and reversed items were adjusted. Scores for RWA were generated by averaging responses to the 12 items. The standardized Cronbach *α* for this scale was 0.81 (*N* = 238).

*Anomie*. In accordance with the Eurobarometer, conducted regularly in Europe, anomie is measured by (a) distrust of institutions, especially political, (b) a feeling that ones' personal situation is deteriorating, and (c) ones' feeling of not being able to control the world around them [28]. Ratings were made on a 6-point scale (1 = strongly disagree, 6 = strongly agree) and individual scores for *Satisfaction*, *Lack of control* and *Distrust of politicians* were computed by averaging the related items (3 items for each dimension), which were shown to be reliable by Wagner-Egger & Bangerter [28]. The standardized Cronbach *α* for satisfaction was 0.67 (*N* = 256), that for lack of control was 0.51 (*N* = 196) and that for distrust towards authorities was 0.88 (*N* = 256).

*Internal Locus of Control*. To measure the extent to which respondents believe they can control the events affecting them we used 5 items from the internality subscale of LOC [28, 54]. Participants were asked to rate the extent to which they agree or disagree with the survey items of a 5-point scale (1 = strongly disagree, 5 = strongly agree). Scores for internal LOC were generated by averaging the 5 items, that proved to be reliable [28]. The standardized Cronbach *α* for internal LOC was 0.71 (*N* = 196).

*Belief in a Dangerous World* We used an abbreviated, 5-item version of the *Dangerous and threatening social world view* scale [55]. Ratings were made on a 6-point scale (1 = strongly disagree, 6 = strongly agree) and individual BDW score were computed by averaging the five items, as in Wagner-Egger & Bangerter [28]. The standardized Cronbach *α* for BDW was 0.81 (*N* = 256).

*Irrationality*. An abbreviated 3-item adaptation of the scale introduced by Wagner-Egger & Bangerter [28] was employed. Respondents were asked to indicate the extent to which they believe in the existence of poorly understood phenomena (astrology, clairvoyance and premonitory dreams), on a 6-point scale. The standardized Cronbach *α* for irrationality was 0.85 (*N* = 238).

*Belief in conspiracy theories*. We used an abbreviated 3-item version of the scale used by Wagner-Egger & Bangerter [28]. The items described prominent conspiracy theories such as J.F Kennedy being not assassinated by a lone gun-man but by opponents, and participants rated the plausibility of the theories on a 6-point scale. The standardized Cronbach *α* for this scale was 0.79 (*N* = 60). This scale was only included in the Swiss sample questionnaire, in order to check whether the Conspiracy view of economics subscale we created (see below) was linked with a more general conspiratory mindset. It was not intended to take place among the predictors of all economic views.

#### Lay perceptions of economics

The focal, economic section of the survey included 14 items, devised for the present study. Each item referred to a well-known economic structure or phenomenon (banks, the stock market inflation, unemployment, etc.) and presented four different views on that topic. The four views were arranged as follows: (A) *The Econ101 view*- a basic, text-book economic description of the topic and its operation, (B) *Government Malfunction view*- attributing failures to the authorities, (C) *Conspiracy view*- reflects a belief that small and powerful groups manipulate the economy, (D) *The Bad Invisible Hand view*- attributes unwanted unwelcome outcomes to the system itself, rather than to specific actors in the economy.

For each item, respondents were asked to rate their agreement with all four views presented on a 6-point scale (1 = strongly disagree, 6 = strongly agree; see sample item below). Individual scores for each cognitive reasoning style were computed by averaging responses to each view across all 14 items. Example of item:

*Stock markets*… *(A)*…*are a necessary tool*, *a mechanism that allows for sophisticated financial activity*, *which is an indispensable component of modern economies (B)*…*have evolved uncontrollably in the past decades*, *and the government is not acting vigorously enough to regulate their activity (C) …are easily manipulated by the select few who can influence it via speculation*, *causing many small players and individuals to lose a great deal of money (D)*…*are an effective way for businesses to develop*, *but it also allows wealthy individuals more power over the economy and over the development of other businesses*. All the items figure in S2 Table.

In order to ensure that the items capture the intended dimension, we performed a validating study using a different sample of Mechanical Turk employees (*N* = 40). For each of the 14 items, we presented participants with four statements reflecting the four different views, and we asked them to match the statements to the view that best describes it. We used a forced choice method. On average, more than 60% of items were classified as intended, against a baseline of 25% of random choice (see S3 Table for details).

**Fig 1:**
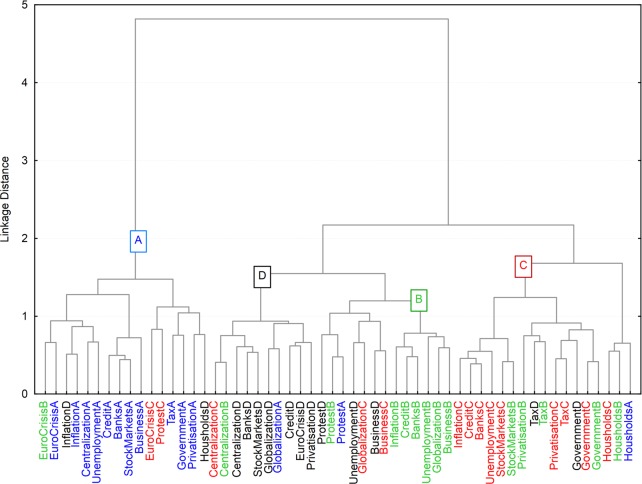
Hierarchical clustering of all the economics questions.

We ran a hierarchical clustering analysis to see to what extent the four views that guided our generation of questions would be found in a purely data-driven analysis. As all our items were on average correlated (total standardized Cronbach *α* = .90), we did not run a more classical Principal Component Analysis, that would artificially produce a number of uncorrelated factors. We used the Pearson correlation for the distance (highly correlated variables are on close branches). Recursive clustering was performed according to Ward's method. This method is a kind of reverse analysis of variance, and attempts to minimize the sum of squares of any two clusters that can be formed at each step [56]. The analysis revealed two main clusters (see Fig 1). The leftmost branch regrouped most of the A questions (11 out of 14 items in the cluster = 69%), along with a minority of other questions labeled for the other views. The other branch split in two. On its right-most branch, furthest away from A, was found a cluster of C questions (9/17 = 53%). The B (7/13 = 54%) and D cluster (7/10 = 70%) split from a common cluster with C. Overall, then, the four views are clearly discernible in the resulting tree, even if the clustering is not totally clean. This is not surprising, as the questions tap into very different economic phenomena (e.g., government, globalization, inflation, banks, etc.) and adherence to each view may vary according to the (group of) topics.

In the following, we use the a priori defined views, whose reliability estimates were high; standardized Cronbach *α* for views A, B, C and D were 0.71, 0.81, 0.86, 0.80 respectively (*N* = 289).

“What happened to the wealth lost to the crisis?” is one context where conspiratorial thinking might express itself. We asked a simple and direct question: *In your opinion*, *what really happened to all the money that was said to have "disappeared" in the recent crisis*? Participants selected one out of the four most common explanations that had emerged in a preliminary survey. Finally, we asked participants to rate whether they were personally affected by the economic crisis on a 4-point scale from 1 = not at all to 4 = strongly (*N* = 204).

## Results

**Fig 2:**
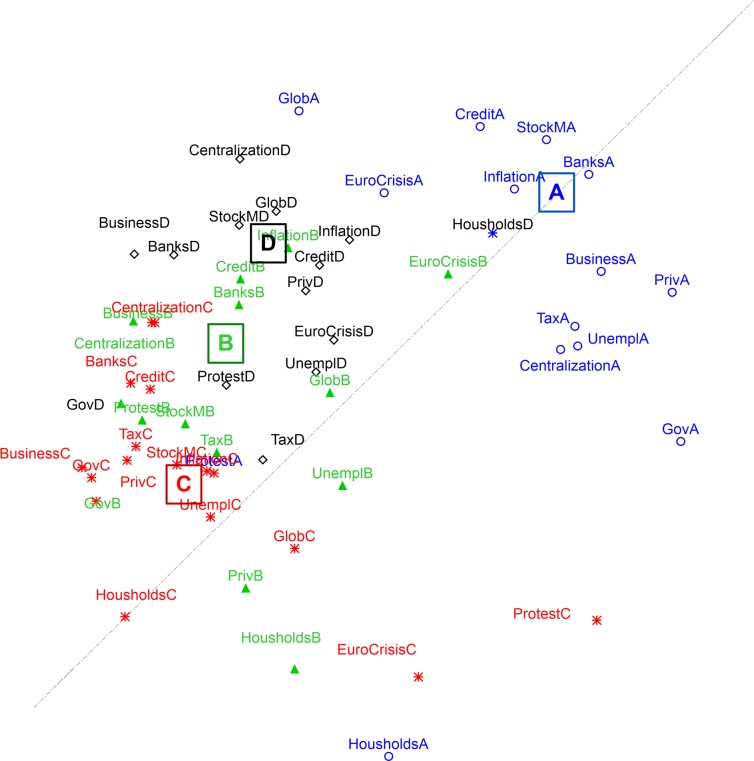
Multi-Dimensional scaling of the items in the Economic scale (proximity expresses Pearson’s correlation) (Stress = .16).

In order to explore the dimensionality of our Economic scale, we performed a Multidimensional Scaling on the intercorrelations between items, that reveals that the four views of economy are fairly distinguishable (see Fig 2). The 3-dimensional solution shows an acceptable stress (.16). The rating of each of the four views about the fourteen different topics cluster quite clearly. The clusters manifest an opposition between the *Econ101* view on the upper right side and the *Conspiracy view* on the bottom left side, the two other views lying in-between, yet closer to the Conspiracy view.

**Fig 3:**
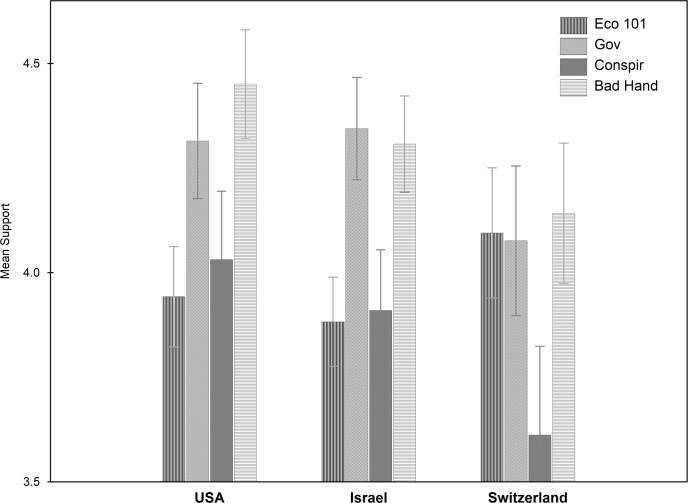
**Means of Views A (Econ101), B (Government Malfunction), C (Conspiracy), D (Bad Invisible Hand) of economy for each country.** (errors bars indicate 95% CI).

The two most and equally popular views are the *Bad Invisible Hand* view (D) and the *Government Malfunction* view (B). These are significantly more endorsed than the other two, the *Econ101* view (A) and the *Conspiracy* view (C), which are also equally popular, *F*(3, 286) = 96.91, *p* < .05, *η*^*2*^_*p*_ = .50 (pairwise Bonferroni post-hoc tests) (see Fig 3). This pattern holds true for all three countries, the only notable exception being a higher mean for the *Econ101* view compared to the other views in the Swiss sample, which is supported to the same extent as the *Bad Invisible Hand* (D) and the *Government Malfunction* views (B).

Pearson correlation coefficients were computed for all variables (Table 2). The correlations were very similar across samples (see S5 Table for details. The only correlation that was reversed in some samples was the correlation between Economic training and the Econ101 view of economics. It was non-significant in the overall sample, but significantly positive in the Israeli sample and significantly negative in the US sample; we do not have an explanation about this result, which should be replicated before being analyzed).

**Table 2:** Pearson correlation coefficients between all variables included in the study.

	1	2	3	4	5	6	7	8	9	10	11	12	13	14	15	16	17	18	19
1. *Econ101* view (A)																			
2. *Government Malfunction* view (B)	.17**																		
3. *Conspiracy* view (C)	-.13*	.70**																	
4. *Bad Invisible Hand* view (D)	.35**	.63**	.54**																
5. Right-Wing Authoritarianism	.23**	-.02	-.07	-.03															
6. Satisfaction	.21**	-.05	-.15*	-.03	.10														
7. Lack of control	-.14*	.24**	.30**	.14*	.16*	-.21**													
8. Distrust of politicians	-.18**	.36**	.46**	.23**	-.04	-.10	.47**												
9. Internal Locus of Control	.28**	.09	-.03	.12	.23	.51**	-.12	-.12											
10. Belief in a Dangerous World	.13*	.33**	.37**	.23**	.39**	-.08	.34**	.39**	-.03										
11. Irrationality	-.17**	-.06	.11	-.10	.08	.06	-.06	-.03	.03	.11									
12. Openness	.06	.09	.04	.08	-.22**	.12	-.20*	-.07	.13	-.08	.06								
13. Conspiracy Theories	.06	.43**	.48**	.13	.25	-.07	.19	.45**	–	.42**	.15	-.05							
14. Age	.01	.10	.08	.05	.11	-.17**	.15*	.12	-.08	.08	-.08	-.12	.03						
15. Sex (1 = men; 2 = women)	.08	.02	-.01	.03	.08	.17**	-.11	-.08	.05	.13*	.25*	.01	.08	-.06					
16. Educational level	.05	-.001	-.04	.02	-.07	-.09	-.05	-.14	-.06	-.14*	-.08	-.16*	–	.01	.01				
17. Religion	-.01	-.09	.03	-.08	.48**	.03	.03	-.03	.06	.18*	.17*	-.13	–	.15*	.24**	-.09			
18. Socio-Economic Status	.06	-.19**	-.22**	-.16*	-.06	.24**	-.07	-.10	-.01	-.15*	-.13	-.05	–	-.09	-.10	.27**	-.17*		
19. Economic training	.05	-.004	-.03	.04	-.06	.02	-.09	-.08	.01	-.06	.05	.02	–	.07	-.18**	.32**	-.01	.16*	
20. Personally affected by the crisis	-.16*	.14*	.17*	.11	.05	-.17	.10	.07	-.01	.07	.18*	-.12	–	.34**	.03	-.15*	.20**	-.38**	.05

Note

* p < .05

** p < .01 (two-tailed tests).

("–" indicates that N = 0, the scales were given to distinct subsamples–see the method section).

**Fig 4:**
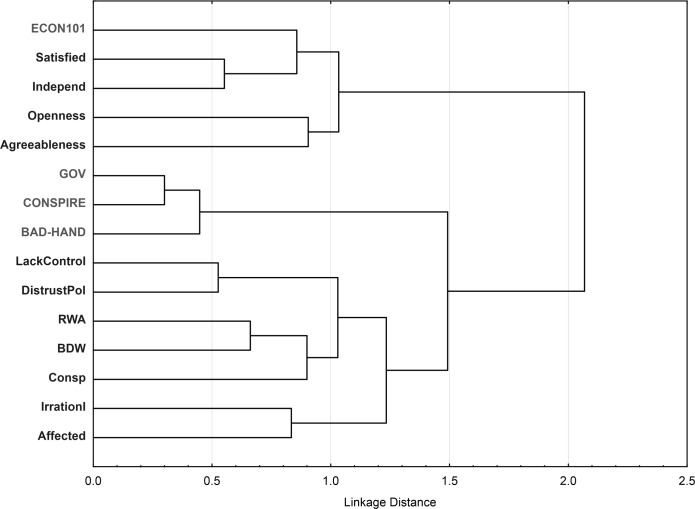
Clustering of variables by their inter-correlation.

To summarize the overall pattern of correlation, a cluster analysis was conducted on the psycho-social scales. Cluster analysis implies two parameters: the definition of distance between items (here, the variables), and the clustering method. We again used the Pearson correlation for the distance (highly correlated variables are on close branches), and Ward's method. Fig 4 shows that the four accounts cluster as in Fig 1 in two groups–*Econ101* lies on one main branch, while the other three accounts are closely clustered together. In terms of the correlations with the other variables, *Econ101* is correlated with Satisfaction with the economic situation, and with Internal Locus of Control. The other three accounts correlate with a cluster comprising all the other variables.

A series of hierarchical regression analyses was run on the four economical views, with socio-demographic (step 1) and psycho-social variables (step 2) as predictors (Table 3). Support for Conspiracy theories was not included in the regressions, because it was a control variable measured in the Swiss sample only.

**Table 3:** Standardized regression coefficients from hierarchical regression analyses with Conspiracy view as criteria, and the socio-demographic variables (step 1) and personality and psycho-social scales (step 2) as predictors.

Criteria Predictors	Econ101 view		Government Malfunction view		Conspiracy view		Bad Invisible Hand view	
	Step 1	Step 2	Step 1	Step 2	Step 1	Step 2	Step 1	Step 2
Age	0.17	0.23**	0.06	0.03	0.01	-0.03	0.01	-0.04
Gender	-0.10	-0.17	0.12	0.12	0.02	0.02	0.01	0.06
Educational level	-0.07	0.05	-0.17	-0.04	-0.08	0.03	-0.08	0.03
Religiosity	0.01	-0.13	-0.09	-0.13	-0.01	0.02	-0.09	-0.15
Socio-Economic status	-0.01	-0.02	-0.18*	-0.18	-0.2*	-0.17*	-0.18	-0.16
Economic training	-0.1	-0.13	0.07	0.05	0.04	0.01	0.07	0.05
Personally affected	-0.13	-0.06	0.09	0.13	0.09	0.09	0.11	0.15
Satisfaction		0.15		0.06		-0.04		-0.06
Lack of control		-0.04		0.07		0.09		-0.02
Distrust of politicians		-0.21*		0.25*		0.32**		0.24*
Right Wing Authoritarianism.		0.14		-0.04		-0.19*		-0.01
Internal LoC		0.19*		0.06		0.03		0.16
Belief Dangerous World		0.28**		0.23*		0.23**		0.20*
Openness		0.09		0.07		0.05		0.09
Irrationality		-0.23**		-0.01		0.19**		-0.05

Note

* p < .05

** p < .01 (two-tailed tests).

The demographic variables did not explain a significant part of the variance of the *Econ101* view (A), *R*^*2*^ = .05, *F*(7, 137) = 1.11, *p* > .05, but adding the psycho-social scales did significantly improve the model, *ΔR*^*2*^ = .26, *F*(8, 129) = 6.06, *p* < .05, and explained a significant part of the variance, *R*^*2*^ = .31, *F*(15, 129) = 3.90, *p* < .05. Age and Belief in a Dangerous World positively predicted the endorsement of *Econ101* view, whereas Distrust of politicians and Irrationality negatively predicted the endorsement of *Econ101* view.

The demographic variables accounted for a statistically significant part of the variance of the *Government malfunction* view (B), *R*^*2*^ = .11, *F*(7, 137) = 2.39, *p* < .05, but adding the psycho-social scales significantly improved the model, *ΔR*^*2*^ = .16, *F*(8, 129) = 3.60, *p* < .05. The whole model explained a significant part of the variance, *R*^*2*^ = .27, *F*(15, 129) = 3.20, *p* < .05. Distrust of politicians and Belief in a Dangerous World positively predicted the endorsement of *Government malfunction* view, whereas Socio-Economic Status negatively predicted the endorsement of *Government malfunction* view.

The demographic variables did not explain a significant part of the variance of the *Conspiracy* view (C), *R*^*2*^ = .07, *F*(7, 137) = 1.53, *p* > .05. Adding the psycho-social scales significantly improved the model, *ΔR*^*2*^ = .29, *F*(8, 129) = 7.43, *p* < .05, and explained a significant part of the variance, *R*^*2*^ = .37, *F*(15, 129) = 4.94, *p* < .05. Distrust of politicians, Belief in a Dangerous World and Irrationality positively predict the endorsement of *Conspiracy* view, whereas Socio-Economic Status negatively predict it.

The demographic variables did not explain a significant part of the variance of the *Bad Invisible Hand* view (D), *R*^*2*^ = .07, *F*(7, 137) = 1.49, *p* > .05, but adding the psycho-social scales again significantly improved the model, *ΔR*^*2*^ = .11, *F*(8, 129) = 2.23, *p* < .05, and explained a significant part of the variance, *R*^*2*^ = .18, *F*(15, 129) = 2.2, *p* < .05. Distrust of politicians and Belief in a Dangerous World positively predicted the endorsement of *Bad Invisible Hand* view (D).

**Fig 5:**
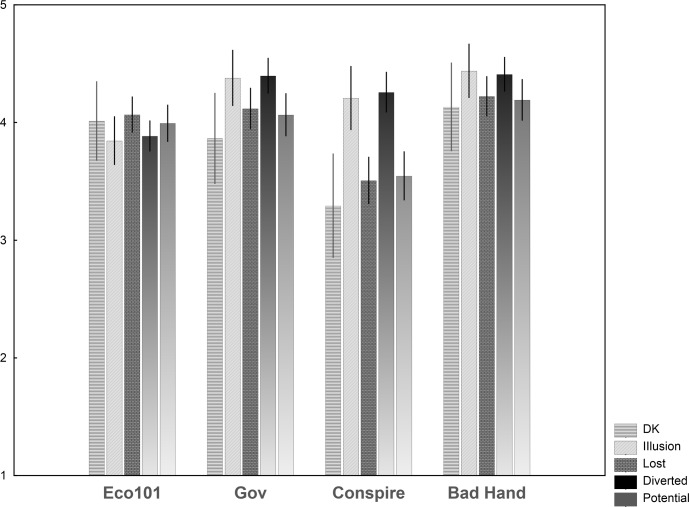
Mean support for each account (Econ101, Government Malfunction, Conspiracy, and Bad Invisible Hand) by explanation selected for "What happened to the money?" (Errors bars indicates 95% CI).

The question on what happened to the trillions of dollars were lost to the crisis is revealing about the nature and the relations between the four views (see Fig 5).

Participants selected, out of four explanations, the one that best matched their own view (or checked the Don't Know answer): (1) It never existed: its existence before the crisis was just an illusion. (2) Shares fell, and as a result part of their value was lost during the crisis. (3) It was not lost for everyone, and is now in the hands of certain people. (4) Its value existed only as a potential, and the crisis destroyed that potential. People who picked answer 1 (it was an illusion) or 3 (the money was diverted) endorse the *Econ101* view (in the main part of the questionnaire) less than those who believe that the money was truly lost, either because shares fell (2) or because the opportunity to transform the potential value into real value was lost (4) (see [57] who discusses the difference laypeople see between the financial "formal economy" and the real economy). The opposite pattern is observed regarding the endorsement of the *Government Malfunction* and the *Bad Invisible Hand* views, and this is even more clearly visible regarding endorsement of the *Conspirational* account. It is also noteworthy that those who Don't Know what happened to the money tend to not support the *Conspirational* account. To our knowledge, this result is the first to demonstrate that–as had been hypothesized by research on conspiracy theories (e.g., [31]–conspiracist ideation may satisfy the need for explanations of distressing phenomena.

## Discussion

This study examined support for four types of accounts in the economic domain over a range of questions: the neo-liberal economic view (which we labeled *Econ101*), the *Government Malfunction* view, *Conspiracy* (destructive outcomes are due to sinister forces who manipulate the system) and the *Bad Invisible Hand* (the invisible hand may go wrong and the equilibrium reached by its doings is not necessarily desirable). All four scales had respectable alphas, indicating that people are consistent in their views regarding economic affairs. Whether discussing tax increases, privatization of public resources, causes of unemployment and inflation, or the role of commercial banks, people hold a rather consistent view. In the case of the conspiratorial mindset, this finding may be seen as an extension of the monological nature of conspiracism [16, 19–21, 23, 24, 26–28, 42, 58, 59]. Moreover, the economic conspiracy view is strongly correlated (in our Swiss sample, the only sample for which this data was collected) with other non-economic conspiracy theories (JFK's assassination, Diana's death, Apollo moon landing).

The pattern of inter-correlations between the four accounts reveals two clusters, *Econ101* and the other three–which fits the perspective identified by [11, 60]. This structure reveals a general opposition between people who trust the neoliberal economic system, and those who mistrust it. The same pattern is observed in the pattern of correlations between the four accounts and the psycho-social variables. *Econ101* is positively correlated with Satisfaction with Life (SWL), Internal LOC, Right-Wing Authoritarianism, and negatively with Lack of control, Distrust of politicians, Irrationality, and Personal harm due to the economic crisis. By contrast, the *Government Malfunction*/*Conspiracy*/*Bad Invisible Hand* views are all positively correlated with Lack of control, Distrust of politicians, moderately with personal harm due to the crisis, and negatively with Beliefs in conspiracy theories and Socio-Economic Status. Belief in a Dangerous World, which may be considered as a measure of the contemporary feeling of insecurity in our modern societies, is positively related to the endorsement of all four economic views. Concerning "classical" Conspiracy Theories, such as those relating to Kennedy's assassination or Lady Diana's death, our study found the usual correlations with anomia (Distrust of politicians; one-tailed test), "social" anxiety (Belief in a Dangerous World; one-tailed test), Right-Wing Authoritarianism, and a positive but non-significant correlation with irrational beliefs (one-tailed tests).

Among the correlates of the four lay economic views, regressions analyses reveal that the strongest (i.e., not mediated) determinants of beliefs are Distrust of politicians (which positively predicts *Econ101* view, negatively the three other views) and Belief in a Dangerous World (which positively predicts the four views of economy). Socio-Economic Status negatively predicts the *Government Malfunction* and *Conspiracy view* (and negatively but non-significantly the *Bad Invisible Hand* view too). Moreover, Internal Locus of Control and age positively predict espousal of the *Econ101* view. Interestingly, Right-Wing Authoritarianism *negatively* predicts the economic *Conspiracy view*, whereas most previous studies found a positive relationship between RWA and classical Conspiracy Theories [19, 28, 38]. Thus, *economic* conspiracy theories would constitute an instance of "leftist conspiracy theories", seldom found in previous research [61].

It has to be stressed that the feeling of insecurity (here measured by the Belief in a Dangerous World) is the only variable that positively and robustly correlates with all four types of economic explanations. This can be explained by the social function of collective representations, such as the views of economy we identified here. Traumatic collective events like economic crises need to be assimilated by the groups that experience them, what can be done by constructing a cause [49]. Built on social representation theory [62, 63], the Collective Symbolic Coping model [64] describes how social groups make sense of the appearance of threats to the social order (i.e., earthquakes, disease outbreaks, etc.), in order to collectively cope with the resulting anxiety. Social coping operates via the construction and diffusion of representations, often through mass media, that enable people to interpret and deal with the new threat. CSC is hypothesized to occur in four consecutive stages. In the *awareness* stage, the new situation emerges as an issue through media agenda setting. Then, intensive communication leads to a *divergence* stage during which multiple explanations emerge, creating ambiguity around the new situation. The third stage is called *convergence*, in which a dominant discourse emerges, lessening the ambiguity of the preceding phase. In the fourth stage, *normalization*, the dominant explanation of the event is integrated into common knowledge. Regarding the economic crisis, we may posit that common knowledge is at the *divergence* stage, because our data suggest the presence of several alternative explanations.

Overall, this set of analyses brings to light a dual view of economy. As was seen in Fig 1, questions relating to each of the four accounts are clustered together, while the views themselves appear to be organized by a split between a *neo-liberal* view of the economy (A) and three critical accounts (B, C, D), that variously attribute the failure of the existing neo-liberal economic system to several compatible accounts. Going from top right to bottom left (in both the political sense and in the figure) the causes are: the neo-liberal view (A- *Econ101)*, and then three critical views: closest to A, failure is part of the functioning of the system itself (D- *Bad Invisible Hand*), that is, the liberal system perspective is accepted, but the respondent is aware that unwelcome and negative consequences can occur too. Further to the left, failures are attributed to the dysfunction of the authorities (B- *Government Malfunction*), or at the other extreme, to powerful groups who manipulate the markets to further their interests (C- *Conspiracy*). The three critical views are held more strongly by individuals from dominated groups of the society (i.e., with a lower socio-economic status), whereas the first, the optimistic, blithe neo-liberal view is advocated by individualist people who fit the dominant ideology (Internal Locus of Control, Satisfaction with Life, etc.).

## Supporting information

S1 Table Psycho-Social Scales. (PDF)

S2 Table Economic Scale. (PDF)

S3 Table Economic Scale Validation. (PDF)

S4 Table Sample Composition for Psycho-Social Scales and Demographics. (PDF)

S5 Table Pearson Correlation Coefficients by Sample. (PDF)

S1 Dataset (XLSX)

S2 Dataset Economic Scale Validation. (XLSX)
