# Supplementary material for: The conspiratorial style in lay economic thinking
Source: PLoS One. 2017 Mar 3;12(3):e0171238. doi: 10.1371/journal.pone.0171238 (PMC5336227; doi:10.1371/journal.pone.0171238)
Supplement: S1 Table — (PDF) [file pone.0171238.s001.pdf]

S1 Table. Psycho-Social Scales.

**Big 5**		*I see myself as someone who...* (degree of agreement on a 6 point scale)
	Agreeableness	…is generally trusting
		…tends to find fault with others*
	Openness	…has few artistic interests*
		…has an active imagination
**Anomie**	*Satisfaction*	*Select the response that best applies to you* (satisfaction on a 6 point scale)
		On the whole, are you satisfied with the life you lead?
		Select the response that best applies to you (Improvement on a 6 point scale)
		If you your present situation with five years ago, would you say it has improved or got worse?
		In the course of the next five years, do you expect your personal situation to improve or get worse?
	Lack of Control	*Please indicate whether you tend to agree or tend to disagree with the statements below* (degree of agreement on a 6 point scale)
		Public services look less and less after the interests of people like me
		There is nothing one can do to change things in our society
		The world is getting worse
	Distrust authorities	*Please indicate whether you tend to agree or tend to disagree with the statements below* (degree of agreement on a 6 point scale)
		The people who run this country are more concerned with themselves than with the good of the country
		Corruption amongst politicians is increasing
		The way government and public bodies work is getting worse
**Right-Wing Authoritarianism.**		*Please indicate to which extent you agree with the following statements* (degree of agreement on a 6 point scale)
		People should develop their own personal standards about good and evil and pay less attention to traditional forms of guidance.*
		What our country really needs instead of more "civil rights" is a good stiff dose of law and order.

		The days when women are submissive should belong strictly in the past. A "women's place" in society should be wherever she wants to be.*
		The withdrawal from tradition will turn out to be fatal one day.
		There is no such crime to justify capital punishment.*
		Obedience and respect for authority are the most important values children should learn.
		Homosexual long-term relationships should be treated as equivalent to marriage.*
		What our country really needs is a strong Prime Minister to lead us.
		It is good that nowadays young people have greater freedom to "make their own rules" and to protest against things they don't like.*
		Being virtuous and law-abiding is in the long run better for us than permanently challenging the foundation of our society.
		It is important to protect the rights of radicals and extremists in all ways.*
		The real keys to the "good life" are obedience, discipline and virtue.
**Internal Locus of Control.**		*Please indicate to which extent you agree with the following statements* (degree of agreement on a 5 point scale)
		I am usually able to protect my personal interests
		When I make plans, I am almost certain to make them work
		My life is determined by my own actions
		I can pretty much determine what will happen in my life
		How many friends I have depends on how nice a person I am
**Trust**		*In daily life, to which extent do you think you can trust…* (completely/ not at all on a 5 point scale)
		… the police
		…the judicial system
**Belief in a Dangerous World.**		*Please indicate to which extent you agree with the following statements* (degree of agreement on a 6 point scale)
		Any day now chaos and anarchy could erupt around us, all the signs are pointing to it
		There are many dangerous people in our society who will attack someone out of pure meanness, for no reason at all.
		Every day as society become more lawless and bestial, a person’s chances of being robbed, assaulted, and even murdered go up and up

		My knowledge and experience tells me that the social world we live in is basically a safe, stable and secure place in which most people are fundamentally good.*
		It seems that every year there are fewer and fewer truly respectable people, and more and more persons with no morals at all who threaten everyone else
**Irrationality**		*Here are several poorly understood phenomena. To what extent do you believe they exist?* (completely believe/ don't believe at all on a 6 point scale)
		Here are several poorly understood phenomena. To which extent do you believe that they exist?
		Knowledge of destiny by divination (clairvoyance)
		Astrology
		Premonitory dreams – dreams that foretell the future
**Personally Effected by the crisis**		*To what extent were you personally affected by the financial crisis?*
		A great deal
		Slightly
		Somewhat
		Not at all
**Attitude- financial crisis**		*In your opinion, what really happened to the money that was said to have "disappeared" in the recent crisis?*
		It never existed; its existence before the crisis was just an illusion.
		The shares fell, and as a result part of their value was lost during the crisis.
		It was not lost for everyone, and is now in the hands of certain people.
		Its value existed only as a potential, and the crisis destroyed that potential.
**Belief in conspiracy theories inventory.**		*Before you are 3 statements concerning historic events. To what extent to you consider these statements likely?* (likely/ unlikely on a 7 point scale)
		Former President of the United States John F. Kenney was murdered in Dallas on November 22nd, 1963. The official investigation concluded that the murderer, Lee Harvey Oswald, acted alone. However, various sources dispute this finding, arguing the assassination was orchestrated by the president's rivals (the CIA, the Cuban opposition, the mafia…)

		On August 31st, 1997, princess Dianna and her partner, Dodi Al-Fayed were killed in a car accident in Paris. The investigation concluded that the couple's driver, Henri Paul, who was inebriated at the time, was responsible for the crash. However, some people (including Dodi's father) claim the princess and her partner were assassinated by the British Secret Service.
		On July 21st, 1969, 3 astronauts from the Apollo space shuttle landed on the moon. Several analysis of the photos published by NASA concluded them to be fake. These sources argue the moon landing was a fraud conducted by the US government in the attempt to gain an edge on the Soviet Union during the cold-war.
