# Supplementary material for: The conspiratorial style in lay economic thinking
Source: PLoS One. 2017 Mar 3;12(3):e0171238. doi: 10.1371/journal.pone.0171238 (PMC5336227; doi:10.1371/journal.pone.0171238)
Supplement: S2 Table — (PDF) [file pone.0171238.s002.pdf]

S2 Table. Economic Scale. Degree of agreement on a 6 point scale.

How have private households and individual consumers contributed to the development of the recent economic crisis?	A	Private individuals did not contribute to the crisis; they consumed within their budget limitation, as they should.
	B	Private individuals did not contribute to the crisis. The government is at fault for not limiting the amount of credit given to private lenders by the banks.
	C	Private individuals did not contribute to the crisis, which was precipitated by a small number of wealthy individuals- these have purchased and invested in a manner that deliberately jeopardized the market in order to profit from the crisis.
	D	Private individuals unintentionally contributed to the crisis. They did so by spending more than they had, and the cumulative effect influenced the economy and caused the crisis.
The government…	A	…regards the citizens' wellbeing as its primary goal; ultimately, they act to assist and promote their quality of life.
	B	...doesn't really care [enough] about the citizens and did not properly regulate the economy as was its duty
	C	…acts as puppets in the hands of wealthy and powerful individuals who promote their interests before those of the people.
	D	…attempts to maximize growth and GNP, and is so doing harms the wellbeing of ordinary people.
The business sector (privately owned businesses of all sizes)…	A	…seeks to increase its profits, as it should; our economic system is based on a competitive business sector which in itself is a positive force.
	B	…seeks to increase its profits and should therefore be monitored closely by the government (to avoid cartels, misuse of public assets etc.)
	C	…acts greedily and willfully caused the economic crises of the last few years (such as the sub-prime crisis in the US)
	D	…seeks to increase its profits, as it should. However its actions have had negative consequences on the national and international economy.
The Socio-Economic protest movement:	A	…is the result of the sorry current economic situation, and counteracts the steadily increasing force of capitalism

	B	…demonstrates that the economic and financial leadership failed to prevent the damages of a shrinking economic growth and the increased life-span
	C	…is fueled by powerful groups who wish to overthrow the current government in order to promote their own financial interests.
	D	…is the result of natural economic processes which inevitably favor big businesses over the lower and middle classes of our society
The recent tax increase...	A	…is the result of a need to accommodate a larger elderly population and a workforce stricken by high unemployment.
	B	…is the outcome of ineffective and careless management of public funds. The taxes are constantly increased to make up for the losses created by the government.
	C	…is the government's way of paying for the huge tax breaks and grants she gives to those wealthy individuals who control it.
	D	…is the result of an inappropriate system of taxation that has not been altered to suit today's society.
The privatization of public resources…	A	…is sometimes the most efficient way to manage those resources. Government monitoring is required but overall private initiatives have more incentive to streamline the management of those assets.
	B	...represent an admission of the state's failure to properly manage and keep resources public as they should be.
	C	...is a calculated process of selling out valuable assets at low costs to private hands, impoverishing the public and making the rich richer.
	D	...is executed by the government for a good reason- to ensure that the resources are managed efficiently. However, in practice this usually results in helping very wealthy individuals.
Globalization…	A	…is the result of the progress in communication and transport, the world is becoming smaller than it was
	B	...should be managed by governments to protect citizens from the consequences of globalization: unemployment, pollution, small businesses going under etc.
	C	...is presented to us as a natural development but is actually orchestrated by businesses who aim to cheapen labor costs and operate in countries with looser laws for their benefit
	D	...is the natural outcome advances in communication transport and technology but it results in unemployment, decrease in salaries etc…

Stock markets...	A	...are a necessary tool , a mechanism that allows for sophisticated financial activity which is an indispensable component of modern economies
	B	…have evolved uncontrollably in the past decades and the government is not acting vigorously enough to regulate their activity.
	C	...are easily manipulated by the select few who can influence it via speculation, causing many small players and individuals to lose a great deal of money.
	D	...are an effective way for businesses to develop but it also allows wealthy individuals more power over the economy and over the development of other businesses.
The increase in unemployment	A	…is the result of the fluctuation of economies- today unemployment is high as a consequence of the crisis, once the economy improves unemployment rates will decrease.
	B	...comes from the government's crippled ability to protect certain sectors and positions. It can be solved by giving proper incentives for businesses to hire more workers.
	C	...is the way in which manufacturers manipulate employees to accept low salaries
	D	...is the result of the naturally occurring globalization; more and more jobs are exported overseas where labor is significantly cheaper.
Centralization...	A	...is inevitable. It is only natural have certain ties between major companies, businessmen and banks, especially in smaller economies or markets.
	B	...should be banned in certain fields such as banking and finance; If they are allowed to exist, one can be sure certain distortions will take place, as the companies will use their power to further their goals.
	C	...enables small and powerful groups to exert their influence in a concerted manner in order to increase their profits while ordinary workers are powerless to defend their share.
	D	...occurs naturally as companies wish to develop and grow beyond their specific narrow field but it results in the gathering of too much power in the hands of specific companies and individuals.
Commercial banks…	A	…are necessary in a corrected developed economy. They allow for businesses as well as private individuals to purchase, save and invest.
	B	…should operate under a much stronger regulation of government establishments (i.e. the central bank) to assure that they only give credit to safe loaners.

	C	…are owned by the rich and the powerful, who use them to ensure that their wealth increases and remains in their hands.
	D	…are by definition businesses, and therefore operate to maximize profits. In doing so, they often take great risks which jeopardize all of our savings.
Euro crisis- the financial crisis in Europe and especially Greece, Spain and Cyprus…	A	…results from the unification of European countries of different financial stabilities, currencies and competitiveness.
	B	…could have been prevented if the European Union had enforced stricter regulations regarding the financial practices of its member countries.
	C	…was provoked by the US, in order to dominate the European economies once the Euro began to look like a threat to the dollar's hegemony.
	D	…is the result of a financial decision to unite by the European member states, in order to allow the European market to grow and become more efficient. In the process, countries with the less financial strength suffer.
Credit and mortgages…	A	…are essential in order to enable people to purchase and consume taking into consideration their predictable future income.
	B	…should be closely monitored in order to prevent abuse and exaggerated profits by the lenders
	C	…are exploited by rich individuals who take risks with the money they barrow, knowing they will not be held accountable for their losses.
	D	…are essential in a properly functioning economy to ensure growth, but in practice they widen the gap between the wealthy and the poor.
Inflation…	A	…comes and goes cyclically. It is a natural, however unpleasant, phenomenon in economic reality
	B	…should be restrained by those responsible for the national economy. It can be held in check if appropriate measures are taken.
	C	…is encouraged by key players who benefit from a workforce desperate to work as a result of a high cost of living.
	D	…is a natural phenomenon in economic reality. However, since wages don't rise as fast as prices, people with low incomes suffer the most from the high cost of living.
