# Supplementary material for: The conspiratorial style in lay economic thinking
Source: PLoS One. 2017 Mar 3;12(3):e0171238. doi: 10.1371/journal.pone.0171238 (PMC5336227; doi:10.1371/journal.pone.0171238)
Supplement: S3 Table — (PDF) [file pone.0171238.s003.pdf]

**S3 Table. Economic Scale Validation.**

The economic section of the survey (available in the supplementary material: S2 Table), was validated using a different sample of Mechanical Turk employees (n=40).

For each of the 14 items, we presented participants with four statements reflecting the four different views, and we asked them to match the statements to the view that best describes it. We used a forced choice method.

The views were explained to participants in the following manner (the view labels were not shown):

(A) *The Econ101 view*- "This is a classic textbook description of the topic and its operation."
(B) *Government Malfunction view*- "The government is responsible for the defects in the functioning of the topic."
(C) *Conspiracy view*- "Small and powerful groups manipulate and are responsible for the failings of the topic."
(D) *The Bad Invisible Hand view*- "No one is to blame, but the system sometimes leads to unwanted economic outcomes."

The results are presented in the following table:

| | **% of the sample who matched the item as intended** | | | | |
|---|---|---|---|---|---|
| | A | B | C | D | **% accuracy per item** |
| **Stock market** | 70.00 | 75.00 | 72.50 | 62.50 | **70.00** |
| **Tax** | 57.50 | 77.50 | 72.50 | 57.50 | **66.25** |
| **Government** | 70.00 | 82.50 | 67.50 | 70.00 | **72.50** |
| **Privatization** | 52.50 | 70.00 | 47.50 | 37.50 | **51.88** |
| **Globalization** | 60.00 | 70.00 | 65.00 | 55.00 | **62.50** |
| **Inflation** | 52.50 | 57.50 | 75.00 | 50.00 | **58.75** |
| **Banks** | 72.50 | 77.50 | 65.00 | 60.00 | **68.75** |
| **Unemployment** | 45.00 | 75.00 | 70.00 | 47.50 | **59.38** |
| **Business** | 75.00 | 70.00 | 62.50 | 60.00 | **66.88** |
| **Credit** | 80.00 | 70.00 | 67.50 | 67.50 | **71.25** |
| **Euro** | 57.50 | 57.50 | 57.50 | 42.50 | **53.75** |
| **Protest** | 37.50 | 47.50 | 45.00 | 30.00 | **40.00** |
| **Households** | 52.50 | 77.50 | 62.50 | 52.50 | **61.25** |
| **Centralization** | 45.00 | 60.00 | 67.50 | 42.50 | **53.75** |

| | | | | | |
|---|---|---|---|---|---|
| **% accuracy per view** | **59.11** | **69.11** | **64.11** | **52.50** | **61.21** |
