# Supplementary material for: The conspiratorial style in lay economic thinking
Source: PLoS One. 2017 Mar 3;12(3):e0171238. doi: 10.1371/journal.pone.0171238 (PMC5336227; doi:10.1371/journal.pone.0171238)
Supplement: S4 Table — (PDF) [file pone.0171238.s004.pdf]

S4 Table. Sample Composition for Psycho-Social Scales and Demographics.

	All	USA	Israel	Swiss
Big 5	238	101	77	60
Right Wing Authoritarianism	238	101	77	60
Satisfaction	256	101	95	60
Lack of control	256	101	95	60
Distrust	256	101	95	60
Internal Locus of control	196	101	95	0
Belief in a Dangerous World	256	101	95	60
Irrationality	238	101	77	60
Conspiracy Theory	60	0	0	60
Age	289	101	128	60
Demographics	229	101	128	0
