# Supplementary material for: The conspiratorial style in lay economic thinking
Source: PLoS One. 2017 Mar 3;12(3):e0171238. doi: 10.1371/journal.pone.0171238 (PMC5336227; doi:10.1371/journal.pone.0171238)
Supplement: S5 Table — (PDF) [file pone.0171238.s005.pdf]

S5 Table. Pearson Correlation Coefficients by Sample.

<u>US Sample.</u>

	1	2	3	4	5	6	7	8	9	10	11	12	13	14	15	16	17	18
1. *Econ101* view (A)																		
2. *Government Malfunction* view (B)	.13																	
3. *Conspiracy* view (C)	-.11	.72**																
4. *Bad Invisible Hand* view (D)	.31**	.65**	.58**															
5. Right-Wing Authoritarianism	.12	-.10	-.16	-.15														
6. Satisfaction	.13	.09	.03	.02	.09													
7. Lack of control	-.03	.33**	.35**	.25*	.22*	-.02												
8. Distrust of politicians	.03	.36**	.35**	.32**	-.04	.08	.56**											
9. Internal Locus of Control	.24*	.17	.06	.14	.16	.48**	-.07	.07										
10. Belief in a Dangerous World	.16	.23*	.29**	.09	.38**	.15	.54**	.32**	.03									
11. Irrationality	-.16	.08	.19	-.05	.13	.13	-.04	-.14	.04	.25*								
12. Openness	.14	.10	.02	.07	-.18	.17	-.23*	-.07	.14	-.11	.04							
13. Age	.08	.14	.04	.04	.16	.02	.03	.06	.01	.01	.13	-.15						
14. Sex	-.14	.02	-.05	-.11	.14	.19	-.03	-.18	-.01	.15	.26*	-.07	.33**					
15. Educational level	-.16	-.17	-.07	-.08	-.03	-.16	-.15	-.19	-.11	-.18	.03	-.19	.16	.05				
16. Religion	-.09	-.09	-.05	-.19	.52**	.05	.07	-.02	.06	.28**	.11	-.13	.25*	.35**	.06			
17. Socio-Economic Status	-.05	-.17	-.17	-.18	.00	.21*	.00	.11	.10	-.08	-.06	-.04	.01	.07	.14	-.03		
18. Economic training	-.27**	-.07	.04	-.03	-.18	-.06	-.13	.03	.07	-.15	.05	-.04	.04	-.25*	.43**	-.13	.12	
19. Personally affected by the crisis	-.14	.12	.07	.10	-.05	-.11	.05	-.04	-.11	-.02	.19	-.13	.26**	.10	.13	.08	-.11	.19

*Note: * $p < .05$; ** $p < .01$ (two-tailed tests).*

Israeli Sample.

	1	2	3	4	5	6	7	8	9	10	11	12	13	14	15	16	17	18
1. *Econ101* view (A)																		
2. *Government Malfunction* view (B)	.26**																	
3. *Conspiracy* view (C)	-.17*	.67**																
4. *Bad Invisible Hand* view (D)	.36**	.69**	.55**															
5. Right-Wing Authoritarianism	.35**	.10	-.05	.11														
6. Satisfaction	.23*	-.12	-.29**	-.06	.35*													
7. Lack of control	-.19	.12	.18	-.02	.01	-.22*												
8. Distrust of politicians	-.39**	.26*	.48**	.05	-.26	-.24*	.42**											
9. Internal Locus of Control	.29**	.04	-.12	.10	.37*	.51**	-.17	-.30**										
10. Belief in a Dangerous World	.04	.44**	.44**	.32**	.18	-.22*	.23*	.37**	-.09									
11. Irrationality	-.30**	-.16	.11	-.08	.13	-.13	.23	.26	-.07	-.11								
12. Openness	.03	.07	.17	.11	-.16	.08	.01	.05	.15	.12	.15							
13. Age	.07	-.02	-.05	-.08	.05	-.31**	-.18	-.03	-.22*	.11	-.13	.06						
14. Sex	.08	.11	.13	.11	.03	.03	.01	.04	.09	.16	.16	.04	-.16					
15. Educational level	.20*	.12	.02	.13	-.10	.01	.06	-.06	.03	-.11	-.16	-.19	-.03	.00				
16. Religion	.03	-.08	.08	-.04	.40**	-.04	-.04	-.08	.01	.04	.21	-.02	-.01	.12	-.15			
17. Socio-Economic Status	.13	-.23*	-.24**	-.12	-.08	.33**	-.10	-.23*	-.02	-.23*	-.11	-.15	-.07	-.18*	.27**	-.18*		
18. Economic training	.20*	.04	-.07	.09	.09	.08	-.07	-.17	-.04	.03	.06	.10	.09	-.14	.26**	.11	.17	
19. Personally affected by the crisis	-.16	.07	.12	.01	.15	-.32*	-.21	.08	.04	.18	.12	-.04	.32**	.01	-.39**	.24*	-.53**	-.05

*Note: * $p < .05$; ** $p < .01$ (two-tailed tests).*

Swiss Sample.

	1	2	3	4	5	6	7	8	9	10	11	12	13	14	15
1. *Econ101* view (A)															
2. *Government Malfunction* view (B)	.10														
3. *Conspiracy* view (C)	.09	.75**													
4. *Bad Invisible Hand* view (D)	.57**	.38**	.32*												
5. Right-Wing Authoritarianism	.36**	-.04	.05	-.04											
6. Satisfaction	.12	-.04	-.08	.08	-.11										
7. Lack of control	.02	.12	.19	-.01	.18	-.30*									
8. Distrust of politicians	.18	.53**	.59**	.34**	.16	.00	.19								
9. Belief in a Dangerous World	.38**	.32*	.41**	.34**	.58**	-.20	.14	.55**							
10. Irrationality	-.22	-.16	.03	-.25	-.07	-.08	.00	.16	.09						
11. Openness	-.13	.24	.04	.23	-.33*	.01	-.16	-.06	-.10	-.08					
12. Conspiracy Theories	.06	.43**	.48**	.13	.25	-.07	.19	.45**	.42**	.15	-.05				
13. Age	-.17	.04	.19	-.03	-.14	.14	.04	-.07	-.05	-.05	-.13	.03			
14. Sex	.35**	-.05	-.12	.29*	.06	.28*	-.08	.16	.18	.14	.04	.08	-.09		
15. Personally affected by the crisis	-.36	.19	.30	-.05	.04	-.17	.42*	.09	.07	.26	-.01	.25	.02	-.07	-.06

Note: * $p < .05$; ** $p < .01$ *(two-tailed tests).*
